# Isothiocyanate from *Moringa oleifera* seeds mitigates hydrogen peroxide-induced cytotoxicity and preserved morphological features of human neuronal cells

**DOI:** 10.1371/journal.pone.0196403

**Published:** 2018-05-03

**Authors:** Mohammed Sani Jaafaru, Norshariza Nordin, Khozirah Shaari, Rozita Rosli, Ahmad Faizal Abdull Razis

**Affiliations:** 1 UPM-MAKNA Cancer Research Laboratory, Institute of Bioscience, Universiti Putra Malaysia, UPM Serdang, Selangor, Malaysia; 2 Department of Biochemistry, Kaduna State University, Main Campus, Kaduna, Nigeria; 3 Department of Biomedical Sciences, Faculty of Medicine and Health Sciences, Universiti Putra Malaysia, UPM Serdang, Selangor, Malaysia; 4 Laboratory of Natural Product, Institute of Bioscience, Universiti Putra Malaysia, UPM Serdang, Selangor, Malaysia; 5 Department of Chemistry, Faculty of Science, Universiti Putra Malaysia, UPM Serdang, Selangor, Malaysia; 6 Laboratory of Molecular Biomedicine, Institute of Bioscience, Universiti Putra Malaysia, UPM Serdang, Selangor, Malaysia; 7 Institute of Tropical Agriculture and Food Security, Universiti Putra Malaysia, UPM Serdang, Selangor, Malaysia; 8 Department of Food Science, Faculty of Food Science and Technology, Universiti Putra Malaysia, UPM Serdang, Selangor, Malaysia; University of PECS Medical School, HUNGARY

## Abstract

Reactive oxygen species are well known for induction of oxidative stress conditions through oxidation of vital biomarkers leading to cellular death via apoptosis and other process, thereby causing devastative effects on the host organs. This effect is believed to be linked with pathological alterations seen in several neurodegenerative disease conditions. Many phytochemical compounds proved to have robust antioxidant activities that deterred cells against cytotoxic stress environment, thus protect apoptotic cell death. In view of that we studied the potential of glucomoringin-isothiocyanate (GMG-ITC) or moringin to mitigate the process that lead to neurodegeneration in various ways. Neuroprotective effect of GMG-ITC was performed on retinoic acid (RA) induced differentiated neuroblastoma cells (SHSY5Y) via cell viability assay, flow cytometry analysis and fluorescence microscopy by means of acridine orange and propidium iodide double staining, to evaluate the anti-apoptotic activity and morphology conservation ability of the compound. Additionally, neurite surface integrity and ultrastructural analysis were carried out by means of scanning and transmission electron microscopy to assess the orientation of surface and internal features of the treated neuronal cells. GMG-ITC pre-treated neuron cells showed significant resistance to H_2_O_2_-induced apoptotic cell death, revealing high level of protection by the compound. Increase of intracellular oxidative stress induced by H_2_O_2_ was mitigated by GMG-ITC. Thus, pre-treatment with the compound conferred significant protection to cytoskeleton and cytoplasmic inclusion coupled with conservation of surface morphological features and general integrity of neuronal cells. Therefore, the collective findings in the presence study indicated the potentials of GMG-ITC to protect the integrity of neuron cells against induced oxidative-stress related cytotoxic processes, the hallmark of neurodegenerative diseases.

## Introduction

Reactive oxygen species (ROS) including hydrogen peroxide (H_2_O_2_) are known by their induction of oxidative stress believed to be linked with various neurodegenerative disease (NDD) conditions including but not limited to amyotrophic lateral sclerosis (ALS), Alzheimer’s disease (AD) and Parkinson’s diseases (PD) [1,2]. It occurs through oxidation of vital cellular biomarkers such as nucleic acids and proteins, crosslinking of membrane constituent and lipids of all kinds within and outside cells [3–5]. Even though a number of cell types considered H_2_O_2_ mitogenic at low concentration [6], it is oxidizable effect at overwhelming quantity often leads to the general cellular damage with consequent death via apoptosis and other processes, affecting the host organs severely [7]. This type of action is largely seen in brain cells due to their high sensitivity, high demand of energy and being the host of many peroxidizable molecules [8,9]. However, accumulation of ROS begins in the neuros prior to clinical detections of signs and symptoms of NDDs particularly AD and PD [10,11]. When that happened, apoptotic mechanism usually switches on to eliminate neurons deemed unbearable [12,13], resulting to severe morphological and functional deficit, leading to progressive decline in cognitive and memory well-being [14,15].

Interestingly, the role of reported plant sourced natural compounds with promising antioxidant and anti-inflammatory activities that prevent or delay the occurrence and progression of NDDs, has been pursuing the interest of many researchers in the quest for additional candidates with better potentials [16–18]. Having said that, Glucomoringin-isothiocyanate (GMG-ITC) was reported to have wide range of biological activities such as anti-inflammatory, anti-oxidant, antimicrobial and antiulcer [19–22]. The GMG-ITC was also reported to attenuate damages in spinal cord injury (SCI) [23], and it could be more promising candidate for neuronal protection. GMG-ITC is a hydrolytic product of a rare glucosinolate called glucomoringin (GMG) isolated from the seed of *Moringa oleifera* commonly known as “horse-radish tree” [20], the most popular among species under genus *Moringaceae* [24]. The hydrolytic reaction is catalysed by β-thioglucoside glucohydrolase (Myrosinase) (EC 3.2.1.147), a specific hydrolytic enzyme that is released as a result of damage in different parts of host plant [25]. In view of the aforementioned potentials of GMG-ITC, we therefore investigated the neuroprotective activity of GMG-ITC against H_2_O_2_-induced cytotoxicity on differentiated human neuronal cells, and assessed the surface ultrastructural and internal morphological features by means of cellular and molecular evidences, for better insight on how the compound work, which could be value added to the existing knowledge of the compound.

## Materials and methods

### Isolation, purification and bioactivation of glucomoringin (GMG)

GMG was isolated from the methanolic seeds extract of *M*. *oleifera* according the stipulated method reported by Rajan et al. [25]. In brief, GMG was isolated using ion exchange chromatography system and purified by gel filtration. The isolated GMG was characterised by means of proton (^1^H), carbon (^13^C) and two dimensional (2D) nuclear magnetic resonance (NMR) spectrometry. The purity of the compound was ascertain through high performance liquid chromatography (HPLC) analysis of desulfo-derivatives in line with ISO 91671 method approved by European union commission regulation, EEC No 1864/90 [26]. Molecular weight of GMG was identified using electrospray ionization (ESI) in positive mode. Additionally, 1 mg of the purely isolated GMG was dissolved in 1 ml PBS at pH 7.2 and incubated with 20 μl myrosinase enzymes (Sigma Aldrich) at 37°C. After 15 minutes of incubation, the GMG produced glucomoringin isothiocyanate (GMG-ITC) which the active compound used in the present study. However, the complete hydrolysis of GMG to GMG-ITC was confirmed by HPLC and LCMS analysis employing sinigrin as internal standard as described by Galuppo et al. [27].

### Cell lines and cell cultures

SHSY5Y cells used in the present study were generously provided by UKM Medical Molecular Biology Institute (UMBI), Universiti Kebangsaan Malaysia Medical Centre, Kuala Lumpur, Malaysia. Due to their neuron like characteristics, the cells could be fully differentiated into neuronal cells by appropriate concentration of retinoic acid (RA), thus, are suitable model for neuroprotection research. The cells were maintained in Dulbecco’s Modified Eagle Media and Hams’ 12 (DMEM/Hams’ F12) in ratio 1:1 (Nacalai, Kyoto, Japan), supplemented with 10% fetal bovine serum (FBS), 1% 2 mM essential amino acid (L-Glutamine), 1% (10000 unit/ml of penicillin and 10000 μg/ml of streptomycin) (Nacalai, Kyoto, Japan), and incubated in 5% CO_2_ and 95% humidified atmospheric air at 37°C.

### Differentiation of SHSY5Y cells

SHSY5Y cell differentiation was performed according to the modified protocol described by Lopes et al. [28]. Briefly, the cells were seeded in 6-wells plates at a density of 1 x 10^5^ cells/well. After 24 hours of incubation, 2mL DMEM/F12 media containing 3% heat inactivated FBS and 10 μM all trans retinoic acid (RA) was added to each well in the dark and kept in 5% CO_2_ incubator at 37°C. The differentiation media was changed daily for a period of seven days. At the end of the experiment, RA induced differentiation was examined under phase contrast using inverted light fluorescence microscope (Zeiss Axio Vert A1, Germany) equipped with image acquisition system (AxioCam MRm, Germany), and multiple images were captured independently. The differentiation was further confirmed by immunocytochemistry assay where expression of neuron specific class III β-tubulin was detected by means of Alexa 488 conjugated antibody.

### Immunocytochemistry (ICC) assay

To further ascertain the differentiation of SHSY5Y cells into full neuronal cells by retinoic acid (RA), ICC was conducted according to the protocol enclosed in the kit as follows: the cells were seeded at 24-well plates at a density of 2 x 10^4^ cells/well and dedifferentiated as described above. The differentiated cells were washed three times with cold phosphate buffer saline (0.01 M phosphate buffer, 0.0027 M potassium chloride and 0.137 M sodium chloride) pH 7.4, at 25 °C followed by incubation with 300 μl fixation solution (4% Paraformaldehyde “PFA”, 1M NaOH and PBS) at 25°C for 30 min and washed with PBS thereafter. Permeation solution (1% Triton X-100 and 99% PBS) and blocking (0.3% bovine serum albumin, 10% goat serum, 10% tween 20 and PBS) solution were incubated with the cells at 25°C for 15 and 30 min, accompanied with washing at each stage. Antibody for Class III β-tubulin (Tuj-1), a cytoplasmic neuron specific protein, was added in a ratio of 1:200 blocking solution with subsequent overnight incubation at 4°C. The cells were washed with PBS in the following day and incubated with Alexa fluorophore-488 secondary antibody conjugate (1:200) in the dark at 25°C for 2 hours. Then the cells were then incubated with nuclear counterstaining dye (DAPI dye) 10 min prior to image viewing under inverted light fluorescence microscope (Zeiss Axio Vert A1, Germany) equipped with image acquisition system (AxioCam MRm, Germany), where multiple images were captured independently.

### Cytotoxicity and cell viability assay

GMG-ITC effect on cell viability and its ability to protect neuron cells against H_2_O_2_-induced oxidative damage coupled with the cytoxicity of H_2_O_2_ were evaluated by means of 3-(4,5-dimethylthiazol-2-yl)-2,5-diphenyltetrazolium bromide (MTT) reduction assay according to the modified protocol reported by Ismail et al. [29]. SHSY5Y cells were seeded at density of 1 x 10^4^ in 96-wells plate and differentiated for seven days as described above. To assess the viability influence of GMG-ITC, the cells were incubated with serially diluted concentration (0.313–10) μg/ml of GMG-ITC for 24, 48 and 72 hours. A twenty microliter (20 μl) of MTT solution was added and the plate was incubated in the dark for 4 hours. Thereafter, the reagent was replaced with 200 μl DMSO to solubilize the formazan formed in the wells. Absorbance was measured immediately at 540 nm using microplate reader (Synergy H1, BioTek, USA). Similar analysis was conducted for H_2_O_2_ cytotoxic effect, in which 1000 μM concentration was serial diluted to 15.63 μM and the optical density was used to evaluate the IC_50_ of H_2_O_2_ used in the present study. Additionally, neuroprotection activity of GMG-ITC was ascertained when the differentiated SHSY5Y cells were pre-treated with serially diluted GMG-ITC (0.313–10 μg/ml) in time-dependant manner prior to four hours challenged by 300 μM (IC_50_) H_2_O_2_ for 4 h, followed by addition of 20 μl and 200 μl of MTT and DMSO reagents respectively. Optical density was measured at 560 nm in all respect, and the experiments were conducted in triplicates under aseptic condition.

### Acridine orange and propidium iodide (AO/PI) double staining

SHSY5Y cells were seeded in 6-well plates at density of 1 x 10^5^ cell/well and differentiated as described above. The cells were pre-treated with GMG-ITC and myrosinase separately for 72 hours and exposed to 300 μM H_2_O_2_ thereafter for 4 hours. After trypsinization, the cells were washed twice and re-suspended in PBS. A mixture of 10 μl propidium iodide (1 mg/ml) and 1 μl (10 mg/ml) acridine orange was combined with 10 μl cell suspension and transferred to glass slide after 15 min incubation at room temperature in the dark. The stained cells were examined under inverted fluorescence microscope (Zeiss Axio Vert A1, Germany) equipped with image acquisition system (AxioCam MRm, Germany). Multiple images were taken independently.

### Flow cytometry analysis

Cellular death was detected using Annexin V-FITC apoptosis detection kit (BD Pharmingen, Japan) according to the protocol enclosed in the kit. Briefly, SHSY5Y cells were seeded in 6-well plate at a density of 1 x 10^5^ cells/well, differentiated and pre-treated with GMG-ITC and myrosinase followed by 4 hours exposure to H_2_O_2_ as described above. The cells were trypsinized, washed twice with PBS, and re-suspended in 1X binding buffer (0.1 M HEPES/NaOH pH7.4, 1.4 M NaCl and 25 mM CaCl_2_). Mixture of 5 μl Annexin V-FITC and Propidium Iodide (PI) each was added to 40 μl cell suspension and incubated for 15 min at room temperature in the dark. A 450 μl 1X binding buffer was added to the stained cells thereafter. The content was vortex, filtered and analysed using flow cytometer (Cyan ADP, Beckman Coulter, Brea, CA, USA) equipped with Summit v4.3 software.

### Scanning electron microscopy (SEM)

SEM was conducted by seeding SHSY5Y cells in T25 ml flasks at a density of 1 x 10^6^ cells/flask and differentiated after attachment as described above. The cells were pre-treated with GMG-ITC and myrosinase separately for 72 hours and challenged with 300 H_2_O_2_ for 4 hours. Upon completion of treatment, the cells were trypsinized and washed with PBS accordingly. In house preparatory guideline for SEM obtainable at the Microscopic Unit, Institute of Bioscience, Universiti Putra Malaysia was followed vehemently. In brief, PBS washed cells were fixed with 4% glutaraldehyde and 1% osmium tetraoxide for 6 and 2 hours respectively, and the cells were washed in between with 0.1M sodium cacodylate buffer three times at the interval of 10 min each. Dehydration with 35, 50, 75 and 95% acetone was performed after discarding the fixatives. The cells were further dehydrated three times with 100% acetone and dried off on a critical dryer for 30 min. The dried pellets were coated with gold particles immediately after mounting and were viewed under scanning electron microscope (JSM 6400, Joel, USA). Multiple images were taken at different magnifications.

### Transmission electron microscopy (TEM)

Likewise, TEM was carried out by seeding SHSY5Y cells in T25 ml flasks at the density of 1 x 10^6^ cells/flask and differentiated after being attached as described above. The cells were trypsinized and washed twice with PBS after GMG-ITC and myrorinase pre-treatment coupled with H_2_O_2_ exposure. The cells were fixed with 4% glutaraldehyde and 1% osmium tetraoxide followed by dehydration using various concentration of acetone as previously mentioned. The cells were also infiltrated with a mixture of acetone and resin in a ratio of 1:1 for 60 min, 1:3 for 120 min and 100% resin overnight. Embedment was carried out by inserting the infiltrated cell in to a resin filled beam capsule. The specimen was cut in to 1 μM thick sections using an ultramicrotome after two days of polymerisation at 60 °C in the oven. Toluidine was employed to stain the sections prior to reducing the thickness of the specimen in to 60–90 nm. After uranyl acetate and lead staining for 15 and 10 min respectively, the thinner sections were viewed under transmission electron microscope (JEM-2100F, Joel, USA). Multiple images were taken at different magnifications.

### Statistical analysis

Data are presented as means ± standard deviation, differences between the means of test and control groups were determined by one-way analysis of variance (ANOVA) with Tukey’s multiple compassion, on Statistical Package for Social Sciences (SPSS) software version 21 (Inc., Chicago, Illinois, USA). 95% level of confidence was considered, thus p<0.05 referred to statistical significance.

## Results

### Differentiation of SHSY5Y cells in to full neurons

**Fig 1:**
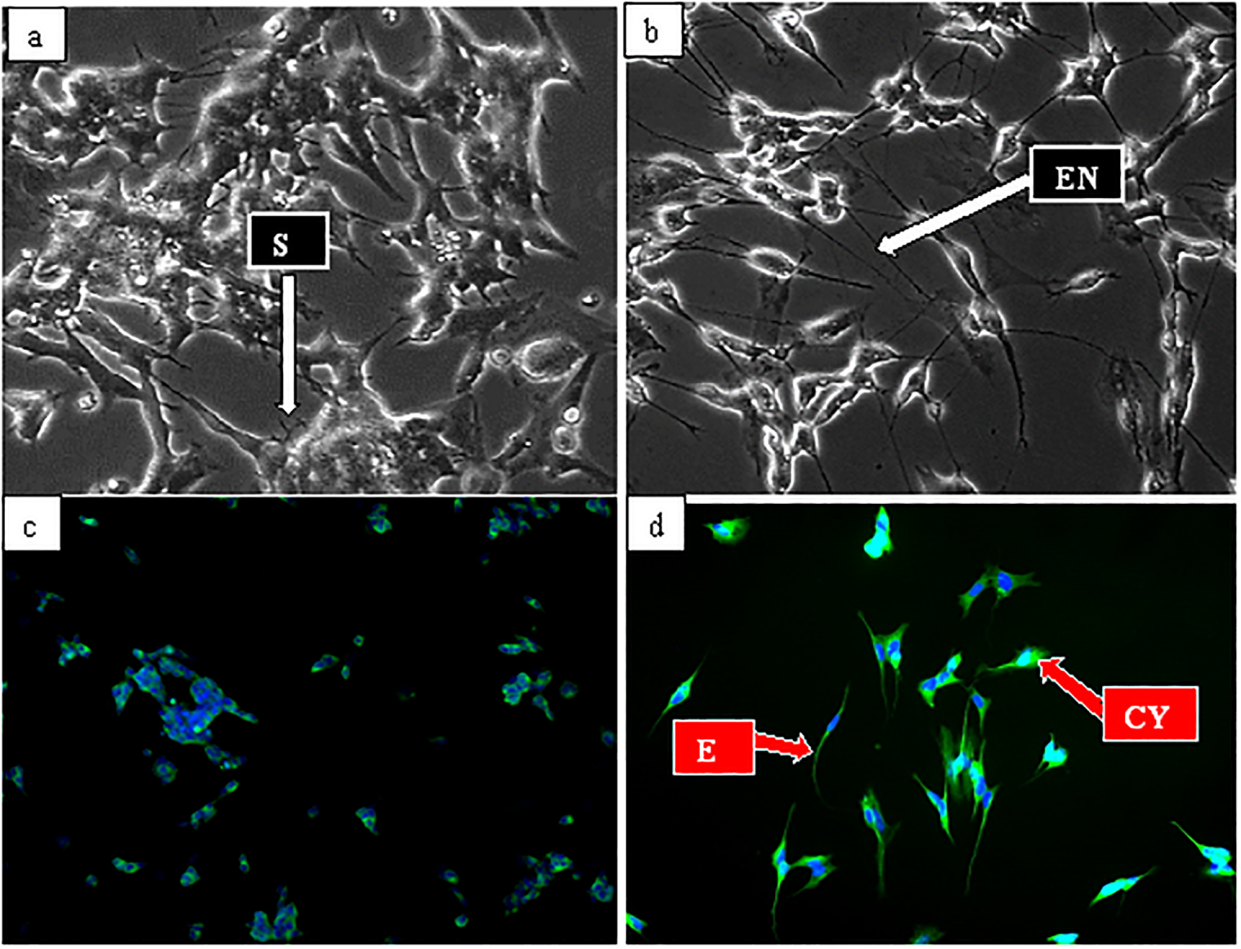
Micrographs of neuronal cells differentiation by 10 μM all trans retinoic acid (ATRA). (a) Undifferentiated cells cultured in 10% complete growth media for seven (7) days and viewed under phase contrast, (b) Differentiated cells cultured in 3% heat-inactivated FBS complete growth media containing 10 μM ATRA for seven (7) days and viewed under phase contrast, and (d) expressed tuj-1 in both cytoplasm and neurites. SN = short neurites, EN = extended neurites, CYP = cytoplasm,. Magnification (x 20).

To demonstrate the transformation of SHSY5Y cells into neuronal lineage used in the present study, the 10 μM retinoic acid (RA) treated cells with extended neurites was observed after 24 hours of treatment (data not shown). The neurite features persisted and intensified after seven days of treatment (Fig 1a). Meanwhile, undifferentiated cells revealed no or comparatively smaller neurites (Fig 1b), indicating that the SHSY5Y cells were differentiated in to typical neuronal cells hence, they were used throughout the experimental analyses.

### Immunocytochemical analysis of neuron specific marker’s expression

The differentiation events of SHSY5Y revealed by phase contrast microscopy was confirmed by immunocytochemistry. Where, fluorescence intensity of the expressed class III β-tubulin (tuj-1) was compared between undifferentiated and seven days RA differentiated SHSY5Y cells. The intensity of green fluorescent appeared weak and detected only in few of the undifferentiated cells (control) (Fig 1c), while that of differentiated increased markedly in both the cytoplasm and neurites (Fig 1d). The increase in green fluorescent intensity indicates high expressions of tuj-1 in the differentiated cells, thus confirming accomplishment of differentiation process.

### Effect of GMG-ITC on H_2_O_2_-induced cell death in neuron cells

**Fig 2:**
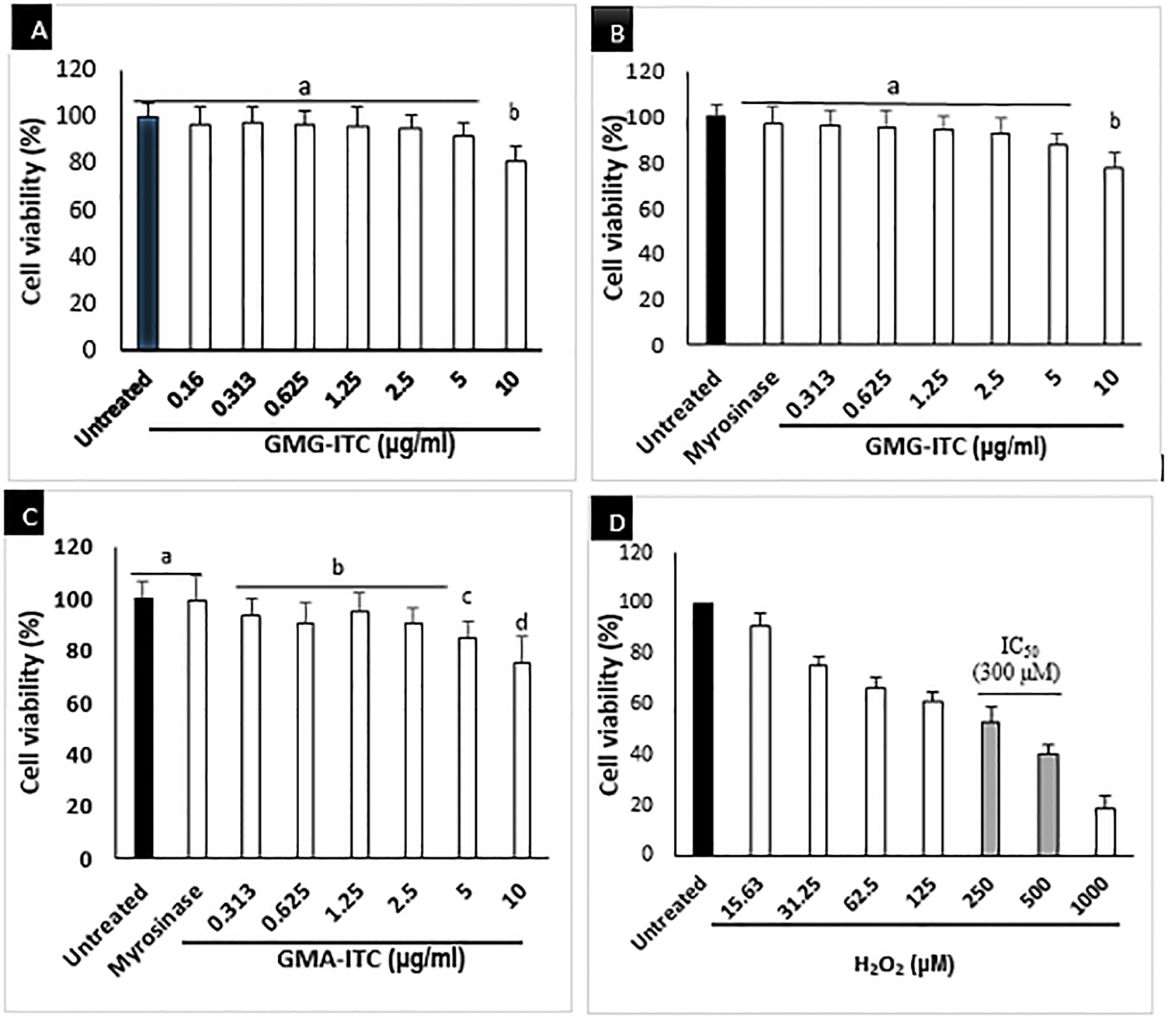
Cytotoxicity of GMG-ITC on differentiated neuronal cells at different concentrations (0.313 to 10) μg/ml. (A) display 24 h, (B) 48 h and (C) 72 h of treatment. Whereas (D) is a cytotoxic analysis result of H_2_O_2_ used in this study with IC_50_ = 300 μM. Values are presented in means ± SD of triplicate experiments and means with different letters varies significantly (p<0.05).

**Fig 3:**
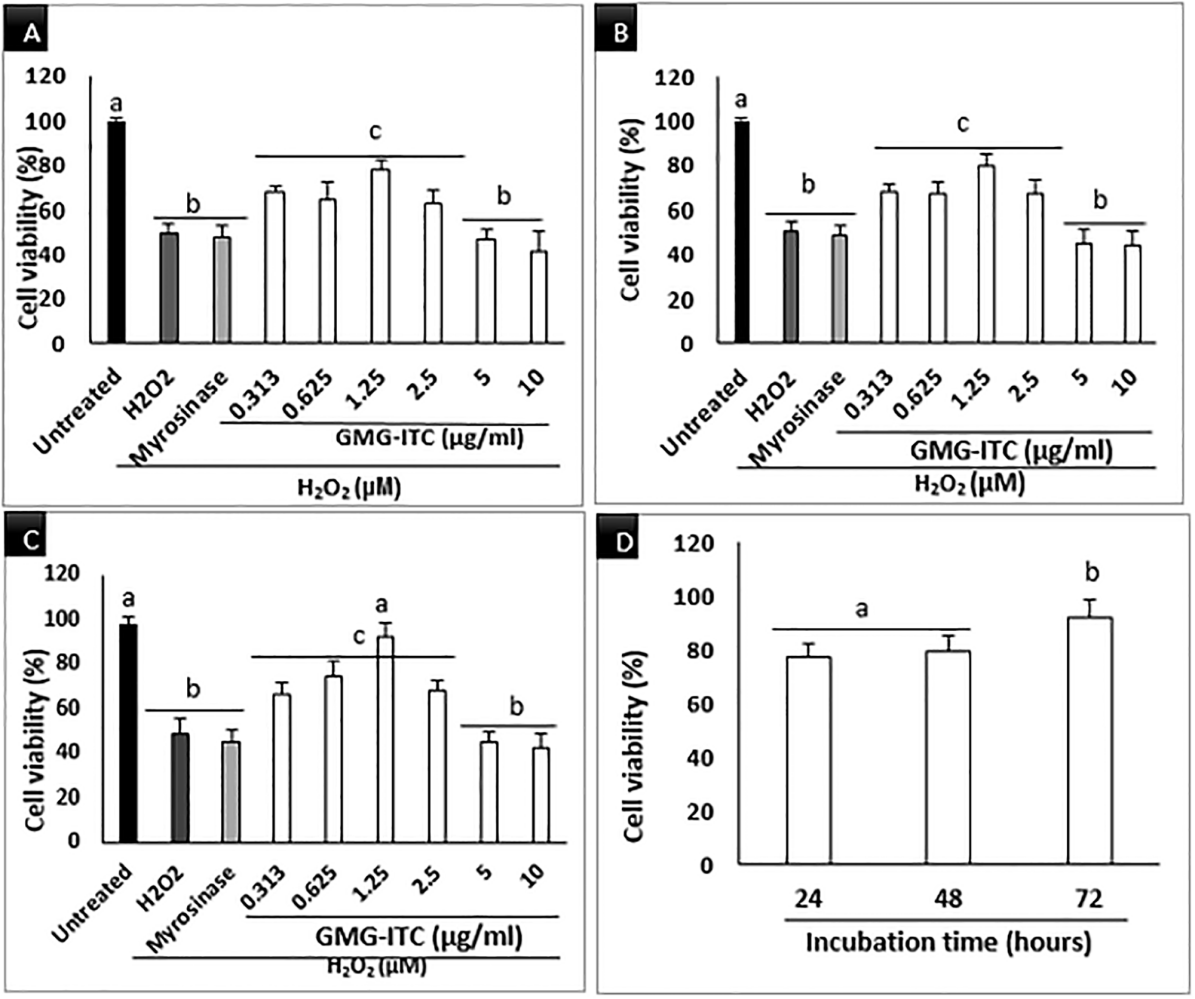
Concentration dependent viability of differentiated neuronal cells, pre-treated with GMG-ITC (0.313–10 μg/mL). (A) 24 h, (B) 48 h and (C) 72 h plus 4 h exposure to 300 μM H_2_O_2_. (D) is a means of 1.25 μg/ml GMG-ITC plus 4 h exposure to 300 μM H_2_O_2_. Values are presented in means ± SD of triplicate experiments and means with different letters varies significantly (p<0.05).

The GMG-ITC treated cells were significantly viable across the concentrations used except those treated with 10 μg/ml, where slight decrease in viability was noticed (Fig 2a, 2b & 2c). on the other hand, the differentiated cells were exposed to H_2_O_2_ at different concentrations (15.6 to 1000) μM in time dependant manner similar to [29], and the result obtained indicated that 300 μM H_2_O_2_ triggered the death of 50% of the cell population in 4 h (Fig 2d). Therefore, it was selected as the concentration of H_2_O_2_ to challenge GMG-ITC pre-treated cells in the subsequent experiments. Also, the GMG-ITC pre-treated and H_2_O_2_ exposed differentiated neuronal cells were analysed accordingly. Although the MTT analysis showed the obvious inhibition of neuronal cells’ viability by H_2_O_2_, pre-treatment of the cells with GMG-ITC provided protection to the cells against the cytotoxic effect of H_2_O_2_ across the experimental period (Fig 3a, 3b & 3c). Interestingly, pre-treatment with 1.25 μg/ml GMG-ITC demonstrated highest viability in all respect especially after 72 hours of treatment (Fig 3d). Hence, it was chosen to be used as working concentration throughout the experiments.

### AO/PI double staining of differentiated neuron cells

**Fig 4:**
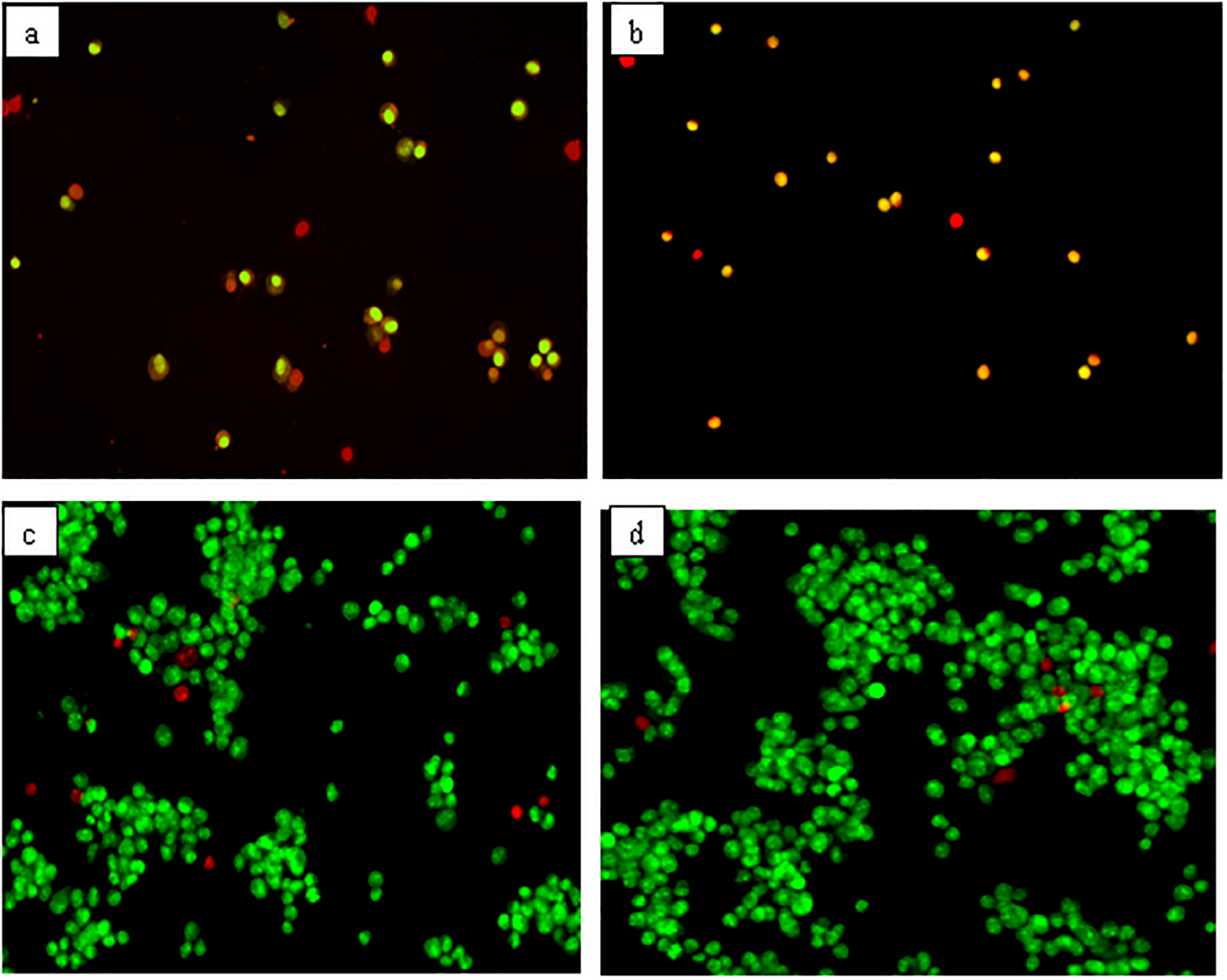
Acridine orange (AO, green) and propidium iodide (PI, red) double staining fluorescent micrographs of differentiated neuronal cells. (a) 4 h H_2_O_2_ treated cells, (b) 72 h myrosinase pre-treated plus 4 h H_2_O_2_ exposed cells, (c) 72 h 1.25 μg/ml GMG-ITC pre-treated plus 4 h H_2_O_2_ exposed cells, (d) untreated cells (normal control). The images were captured in multiple times and x20 magnification was used.

Observation of the differences between GMG-ITC pre-treated and untreated control differentiated neuron cells by means of AO and PI dyes was performed on fluorescence microscope. Green stained nucleus of the cells (Fig 4a–4d) signified viability of the cells, whereas those stained red in the same figures were unprotected against H_2_O_2_-induced cytotoxicity indicating the symbol of apoptosis. High percentage of the GMG-ITC pre-treated cells (Fig 4c) were stained green revealing normal appearance of healthy viable cells.

### GMG-ITC protected differentiated neurons against H_2_O_2_-induced apoptosis

**Fig 5:**
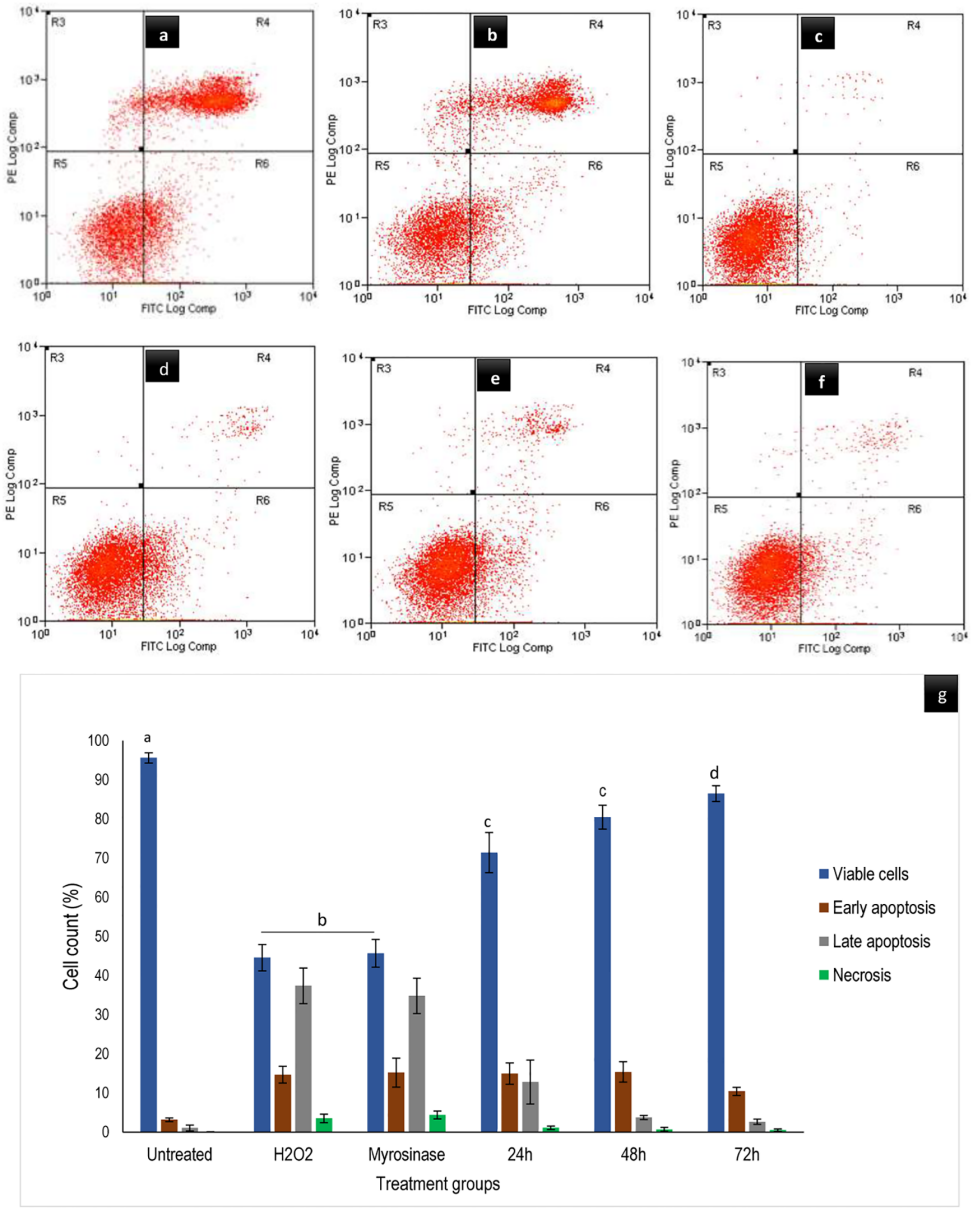
Annexin V-FITC assay of differentiated neuronal cells analysed by flow cytometry. Where (a) 4 h H_2_O_2_ treated cells, (b) 72 h myrosinase pre-treated plus 4 h H_2_O_2_ exposure cells, (c) untreated (normal control) cells, (d) GMG-ITC pre-treated for 24 h plus 4 h H_2_O_2_ exposure, (e) GMG-ITC pre-treated for 48 h plus 4 h H_2_O_2_ exposure and (f) GMG-ITC pre-treated for 72 h plus 4 h H_2_O_2_ exposure. Whereas (g) represent distribution of cells at death. Values are presented in means ± SD of triplicate experiments and means of viable cells with different letters varies significantly (p<0.05).

The results of flow cytometry analysis by means of annexin V-FITC and PI stains for apoptosis evaluation was obtained after GMG-ITC pre-treatment and H_2_O_2_ exposure. It was indicated that, the cells in left-lower quadrant (Annexin-V^-^/PI^-^) appeared to be healthy, those in the right-lower quadrant (Annexin-V^+^/PI^-^) seemed to underwent early apoptosis, and late apoptosis was seen in the right-upper quadrant (annexin-V^+^/PI^+^). Meanwhile necrotic process was observed in the left-upper quadrant (annexin-V^-^/PI^+^) of dot plot (Fig 5a–5f). In comparison to GMG-ITC time dependant pre-treated plus H_2_O_2_ exposure cells, percentage of apoptosis appeared to be much higher than that of necrosis in myrosinase pre-treated (enzyme control) and H_2_O_2_ alone (control) exposed cells (Fig 5g). Also, pre-treatment with 1.25 μg/ml GMG-ITC significantly lowered the early and late H_2_O_2_-induced apoptotic process with remarkable increase in the cells’ viability similar to what was observed in the untreated control cells seen in the same figure (Fig 5g).

### Surface morphological assessment of GMG-ITC pre-treated differentiated neuronal cells

**Fig 6:**
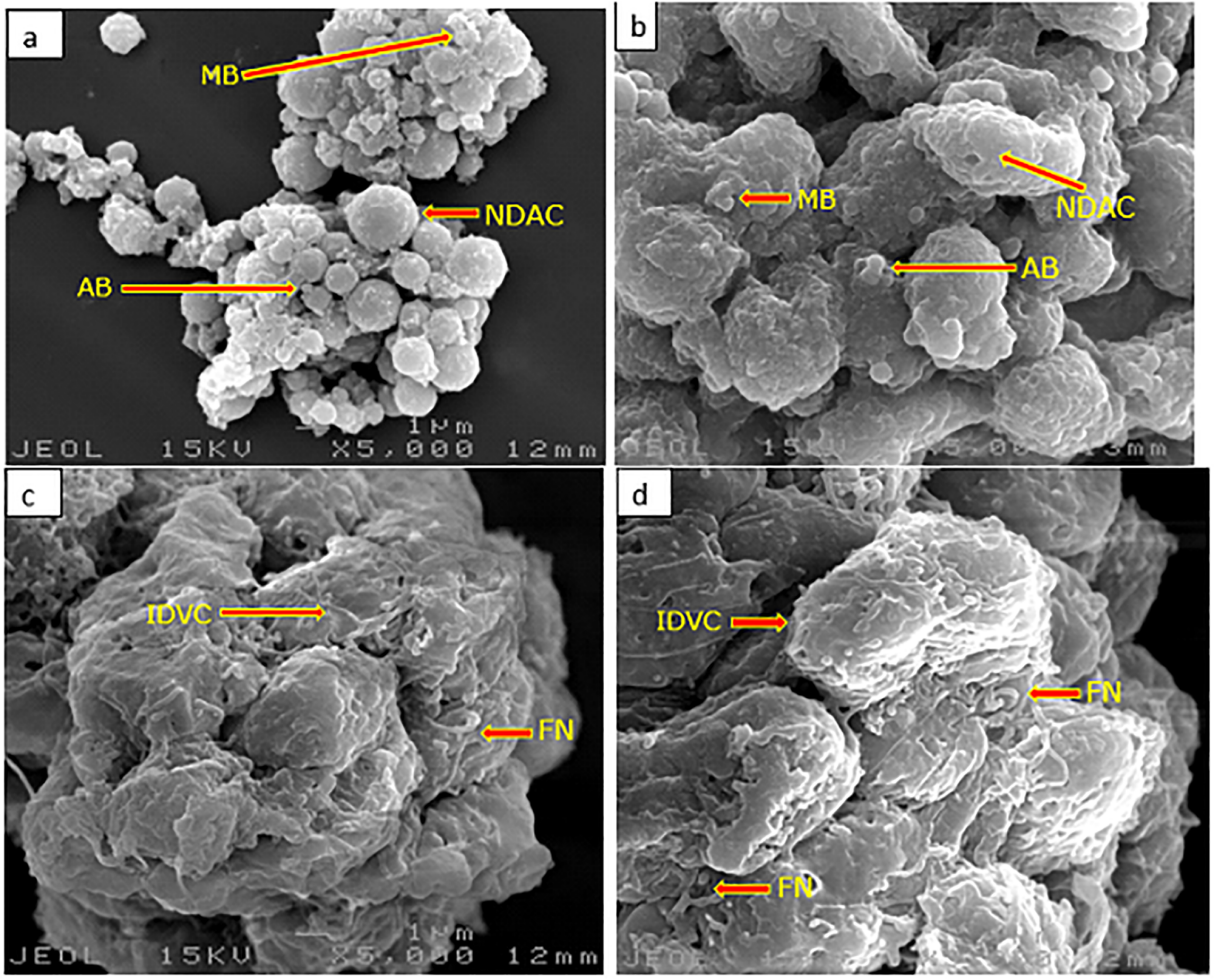
Surface morphological analysis of differentiated neuronal cells by scanning electron microscopy. (a) 4 h H_2_O_2_ treated cells, (b) 72 h myrosinase pre-treated plus 4 h H_2_O_2_ exposure cells, (c) 72 h GMG-ITC pre-treated plus 4 h H_2_O_2_ exposure cells, (d) untreated (normal control) cells. AB = apoptotic body, IDVC = intact differentiated viable cells, FN = folded neurites, MB = membrane blabbing, NDAC = neurite disrupted apoptotic cells. Magnification (x 5000).

Cellular surface ultrastructural analysis of differentiated neuronal cells pre-treated with or without GMG-ITC plus H_2_O_2_ exposure observed on SEM revealed an interesting outcome. Where neurites disruption, membrane blebbing and cell shrinkage were noticed on H_2_O_2_ exposed (Fig 6a) and myrosinase pre-treated plus H_2_O_2_ exposed cells (Fig 6b). However, when the cells were pre-treated with GMG-ITC prior to H_2_O_2_ exposure, their surfaces features appeared intact with folded neurites and integrated cytosol (Fig 6c). The result was similar to the untreated normal control cells seen in Fig 6d.

### Ultrastructural analysis of GMG-ITC treated differentiated neuron cells

**Fig 7:**
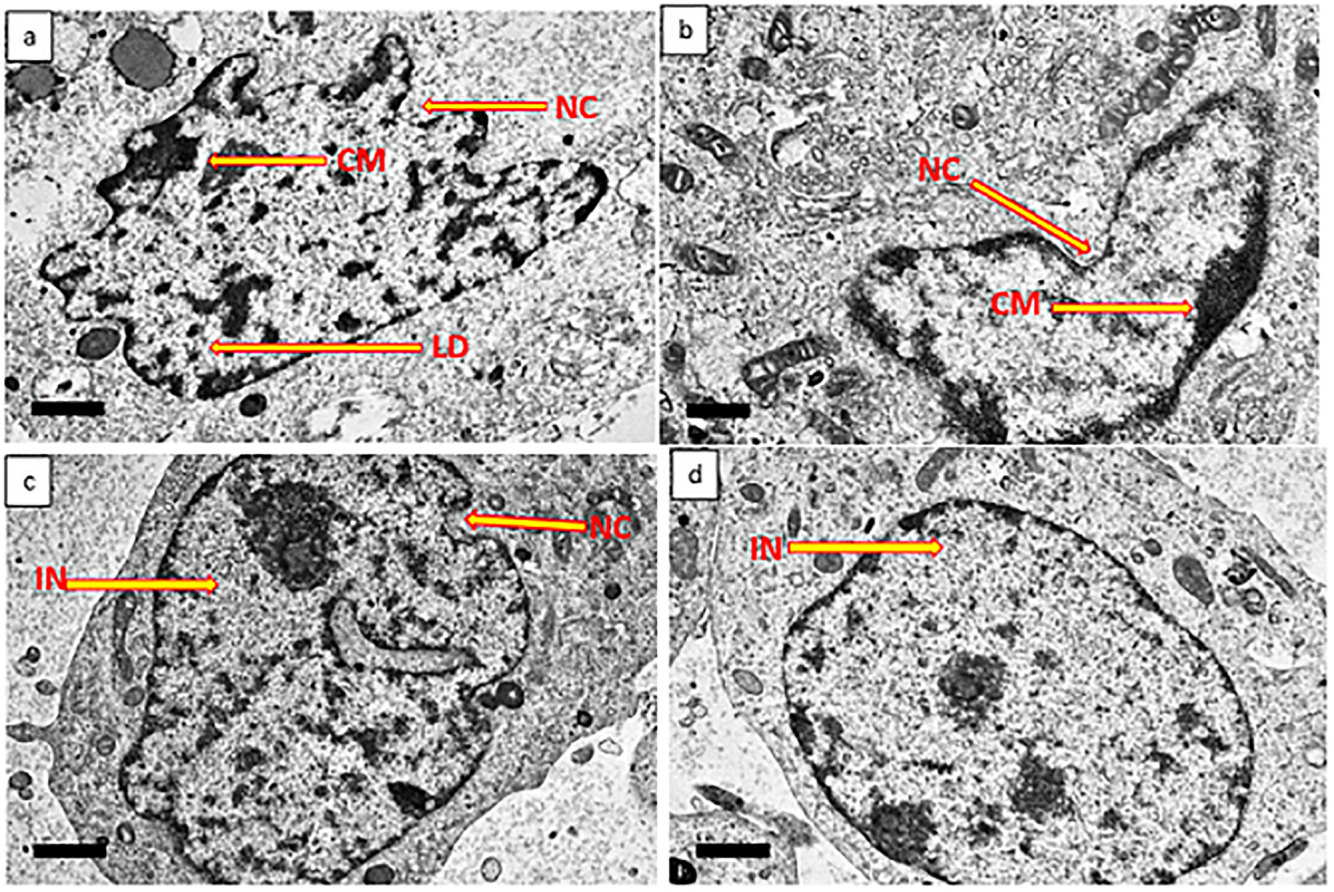
Ultrastructural analysis of differentiated neuronal cells by transmission electron microscopy. (a) 4 h H_2_O_2_ treated cells, (b) 72 h myrosinase pre-treated plus 4 h H_2_O_2_ exposure cells, (c) 72 h GMG-ITC pre-treated plus 4 h H_2_O_2_ exposure cells, (d) untreated (normal control) cells. CM = chromatin margination, IN = intact nucleus, LD = lipid droplet, NC = nuclei convolution. Magnification (x 3000).

The ultrastructural assessment of differentiated neuronal cells performed by means of TEM showed morphological aberration in GMG-ITC untreated but H_2_O_2_ exposed cells. Where nuclear shrinkage, nuclear convolution, chromatin condensation and chromatin margination were obvious (Fig 7a). These features were absent in GMG-ITC pre-treated plus H_2_O_2_ exposure cells (Fig 7c) and untreated (normal control) cells (Fig 7d). However, the cells pre-treated with myrosinase prior to H_2_O_2_ exposure (Fig 7b) demonstrated similar features with H_2_O_2_ alone exposed cells. Again, indicating zero effect of the enzymes in neuroprotection against H_2_O_2_–induced cytotoxicity.

## Discussion

The present study revealed novel insight on neuroprotection ability of an isothiocyanate of *Moringa oleifera* origin against H_2_O_2_-induced oxidative stress. *Moringa oleifera* is a medicinal plant known by many herbalists and folk medicine practitioners as “miracle tree” [30,31]. It is widely used for particularly human consumption and other domestic activities including water purification, in sub-Saharan Africa and other tropical regions worldwide [32]. Due to its richness in naturally occurring compounds, *M*. *oleifera* exhibited numerous biological and health benefits such as anti-inflammation, anticancer, antidiabetic, wound healing and antimicrobial [30,32–34]. The seeds part of the plant contains large quantity of GMG-ITC and its precursor that was reported to prevent oedema with consequent brain damage in transgenic rats [35,36]. Being a dopaminergic neuronal cell, human neuroblastoma cells (SHSY5Y) are becoming more popular as a model for neuroscience research particularly neurodegenerative diseases including but not limited to Alzheimer’s disease (AD), Parkinson’s disease (PD), Huntington’s disease (HD), Amyotrophic lateral sclerosis (ALS) and Multiple sclerosis (MS) [37–39]. The cells develop neuronal properties such as neural extension and expression of certain neuron specific markers upon regular incubation with 10 μM all-trans retinoic acid (RA) or other differentiation inducers for appropriate period [40,41]. However, study have shown that differentiation process of SHSY5Y to full neuronal cells lower their susceptibility to cytotoxic effect of various compounds [42], thereby enhancing their stability compared to undifferentiated version of cells. H_2_O_2_-induced cytotoxicity in differentiated neuron cells resulted in cascade of reactions that overwhelmed endogenous defensive mechanism system of the cells leading to oxidative conditions with consequent cell death [43]. Although, exogenous antioxidants prevent oxidative damage by banishing ROS generation in the cells thereby increasing their chance of survival [44]. Fig 2 above showed how various concentrations of GMG-ITC enhanced viability of differentiated neurons in time dependent manner. However, 1.25 μg/ml GMG-ITC exhibited maximum potential in that respect, signifying high capability for reducing susceptibility incurred by hermetic response in the cells. The effect was obviously higher after 72 hours of treatment compared to 48 and 24 hours. This attribute to long time effect on endogenous defensive mechanism that again reduce the vulnerability of the cells to certain attacks by exogenous cytotoxic agents. Growing number of studies revealed that under normal circumstance, oxidative damage causes reactions that confer negative effect on beneficial markers in antioxidant mechanistic pathways responsible for neutralising harmful stimuli [45,46]. However, exogenous antioxidants tend to counteract such effects, but when presence in high quantity they inhibit the response generated by their indigenous counterpart thereby increasing the cells’ sensitivity to stimuli with eventual death [44].

The enhancing effect of GMG-ITC on cells’ survival was evaluated by means of AO/PI double staining, and it strengthened the earlier claim of GMG-ITC protective effect on differentiated neuron cells. Being permeable to cellular membrane, AO stains cellular nucleus green, revealing viability of the cells. Whereas the PI which is a membrane impermeable intercalating agent could only be taking up by cells with disrupted membrane, thus stained their nucleus red [12,47]. The green stained nucleus (Fig 4) indicated level of protection provided by GMG-ITC pre-treatment prior to cytotoxic induction by H_2_O_2_ that affect some of the cells (stained red or orange) observed in the same figure. Therefore, GMG-ITC demonstrated high neuroprotective activity against cellular death due to H_2_O_2_ exposure.

Furthermore, pre-treatment of GMG-ITC prevented differentiated neuronal cells against early and late apoptosis or necrosis induced by H_2_O_2_ exposure as observed in Fig 5. This indicates definite ability of the compound to keep lipid asymmetry membrane intact. Thus, preventing translocation of phosphatidylserine (PS) to cytoplasm. Although, study have shown that when cells are exposed to oxidative stress conditions, the internally generated ROS promote the disruption of membrane asymmetrical status, causing translocation of PS [48]. This effect may translate through receptor activating signals to break mitochondrial membrane potentials and trigger the release of cytochrome C with consequent cell death via apoptosis [49]. Likewise, the outcome of annexin V-FITC analyses signified that apoptosis is a predominant event occur in H_2_O_2_-induced differentiated neuron cell death. Therefore, GMG-ITC inveterate to be potential anti-apoptotic agent against H_2_O_2_-induced neuronal cell death. Also, record has it that cytotoxicity resulted in devastative cellular morphological changes such as membrane blebbing and cell shrinkage [50]. Ultrastructural surface analysis of the differentiate cells conducted by means of scanning electron microscopy (SEM) demonstrated the ability of GMG-ITC to preserve membrane integrity and protect cell surface structures including extended neurites of differentiated neurons (Fig 6c). Even though, the folded neurites on GMG-ITC pre-treated cells are highly similar to those of untreated cells, the neurites seemed to be disrupted on H_2_O_2_ exposed cells without GMG-ITC pre-treatment. On the contrary, pre-treatment with myrosinase prior to cytotoxic induction offered no effect on the differentiated cells, indicating that the observed neuroprotection against H_2_O_2_-induced oxidative damage is solely provided by GMG-ITC. This affirm our earlier claims on cell viability enhancement potential of GMG-ITC. Additionally, nuclear shrinkage, chromatin condensation and margination are typical apoptotic features in cells undergoing apoptosis [50]. As GMG-ITC prevents the occurrence of such events, we therefore postulate that the compound possessed robust neuroprotection capacity through the abolishment of internal ROS generation mechanisms.

## Conclusion

In the present study, our findings highlighted the increase in viability of differentiated neuron cells in the presence of H_2_O_2_ due to GMG-ITC pre-treatment. Which perhaps facilitated through anti-apoptotic activity of the compound observed on fluorescence microscope and flow cytometry analysis. Interestingly, the result also demonstrated that GMG-ITC is capable of conserving membrane and internal structural integrity of differentiated neurons despite the exposure to oxidative damage by H_2_O_2_, indicating its strength in protecting neurons from degeneration due to oxidative stress. Therefore, this study worth expansion to obtain more evidence on how the compound provides such actions and the actual modulatory mechanistic pathways involved in the process.
